# A tale of two phenotypes: transition from hypertrophic to dilated cardiomyopathy in Danon disease

**DOI:** 10.1093/ehjcr/ytae445

**Published:** 2024-08-26

**Authors:** Xuantong Yue, Kai Yang, Minjie Lu

**Affiliations:** Department of Magnetic Resonance Imaging, Fuwai Hospital and National Center for Cardiovascular Diseases, Chinese Academy of Medical Sciences and Peking Union Medical College, Beilishi Road No. 167, Xicheng District, Beijing 100037, China; Department of Radiology, Sichuan Science City Hospital, Mianshan Road No. 64, Mianyang, Sichuan 621000, China; Department of Magnetic Resonance Imaging, Fuwai Hospital and National Center for Cardiovascular Diseases, Chinese Academy of Medical Sciences and Peking Union Medical College, Beilishi Road No. 167, Xicheng District, Beijing 100037, China; Department of Magnetic Resonance Imaging, Fuwai Hospital and National Center for Cardiovascular Diseases, Chinese Academy of Medical Sciences and Peking Union Medical College, Beilishi Road No. 167, Xicheng District, Beijing 100037, China

A 25-year-old woman with cardiac hypertrophy and Wolff-Parkinson-White syndrome sought diagnosis and treatment at our hospital. The initial electrocardiogram confirmed Wolff-Parkinson-White syndrome (*Figure 1A*), while the echocardiogram revealed hypertrophic non-obstructive cardiomyopathy, with a maximum ventricular septum thickness of 19 mm (*Figure 1B*) 6 years ago. Last year, she returned to our hospital due to chest tightness and dyspnea. Her NT-proBNP level was 6625 pg/ml, and cTnI was 0.42 μg/l. Both electrocardiogram and Holter monitoring identified atrial fibrillation, frequent ventricular premature beats (9261 beats/24 h), and non-sustained ventricular tachycardia. Subsequent echocardiography indicated an enlarged heart, reduced biventricular systolic function, and a notable decrease in ventricular septal thickness to 9 mm (*Figure 1C*). Cardiac magnetic resonance imaging further validated these morphological and functional abnormalities. The left and right ventricular ejection fractions were markedly reduced to 27% and 15%, respectively (*Figure 1D–F*, Supplementary material online, *Videos S1–S3*). Delayed enhancement imaging revealed extensive myocardial enhancement in biventricular walls, with relatively mild involvement of the ventricular septum (*Figure 1G–I*). Both T1 mapping (*Figure 1J* and *K*) and extracellular volume fraction (*Figure 1L*) were elevated. These findings led to the consideration of a decompensation phase in inherited metabolic cardiomyopathy. Genetic testing confirmed the diagnosis of Danon disease with a LAMP2 c.733C > T (p.G245*) mutation (*Figure 1M*). The patient ultimately underwent a heart transplantation and was in good condition at the 3-month follow-up. Danon disease, a rare X-linked dominant lysosomal disorder, predominantly manifests as myocardial hypertrophy, though some patients, particularly females, may develop a dilated phenotype. This case illustrates that Danon disease with a dilated cardiomyopathy phenotype might represent a decompensated or end-stage form of the disease in patients initially presenting with a hypertrophic cardiomyopathy phenotype.

**Figure 1:**
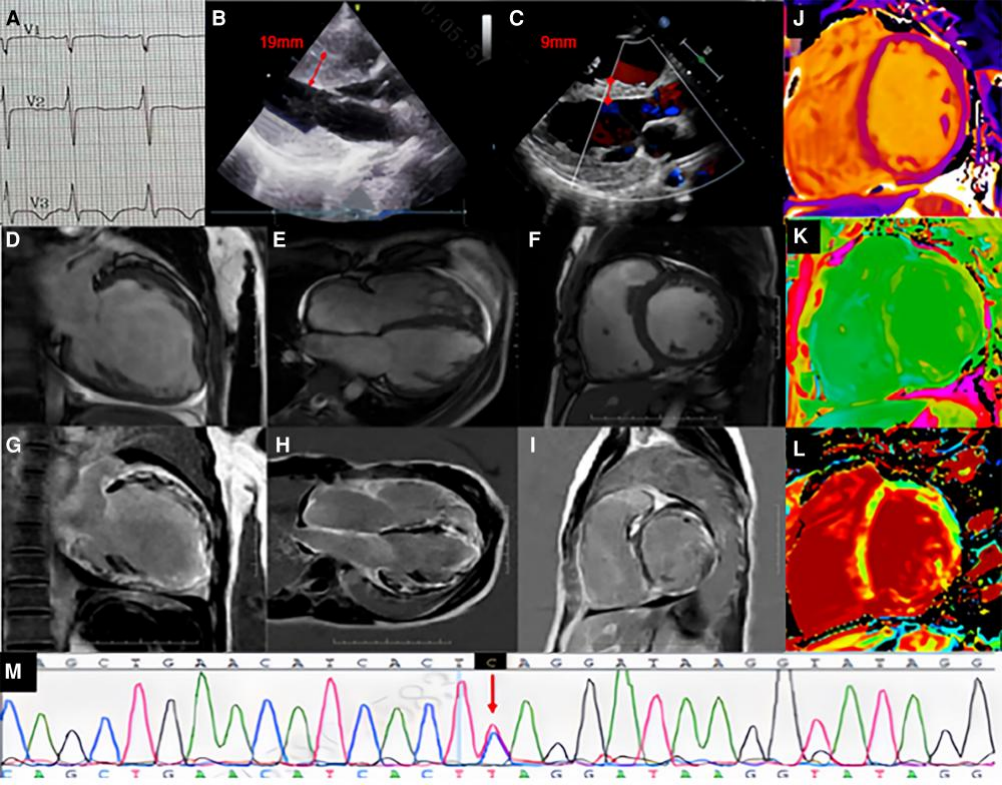
The relevant test results for this patient.

## Supplementary Material

ytae445_Supplementary_Data

## Data Availability

The data underlying this article are available in the article and in its online Supplementary material.

## Supplementary material

**Consent:** The authors confirm that written informed consent for submission and publication of this case report, including images and associated text, has been obtained from the patient in line with COPE guidance.

**Funding:** None declared.

